# Parental burnout at different stages of parenthood: Links with temperament, Big Five traits, and parental identity

**DOI:** 10.3389/fpsyg.2023.1087977

**Published:** 2023-03-31

**Authors:** Konrad Piotrowski, Agnieszka Bojanowska, Dorota Szczygieł, Moïra Mikolajczak, Isabelle Roskam

**Affiliations:** ^1^Center for Research on Personality Development, SWPS University, Poznań, Poland; ^2^SWPS University, Warsaw, Poland; ^3^SWPS University, Sopot, Poland; ^4^Psychological Sciences Research Institute, University of Louvain (UCLouvain), Ottignies-Louvain-la-Neuve, Belgium

**Keywords:** neuroticism, conscientiousness, briskness, endurance, identity, exhaustion

## Abstract

The study aimed to analyze the links between traits from different levels of personality organization and parental burnout. To answer the research questions, a cross-sectional study was conducted with 1,471 parents aged 19 to 45 years (mean age 35.30, SD = 5.98). The results showed that the severity of parental burnout was linked to traits ranging from biologically determined temperament traits to basic personality traits to a sense of parental identity. More specifically, we found higher burnout among parents who have difficulty shifting between tasks and coping with strong stimulation, low emotional stability and conscientiousness, and low identification with the parental role. We also found that certain personality traits were more strongly associated with parental burnout among those who had children in early childhood or preschool period (under the age of seven) than those in later stages of parenthood. The study contributes knowledge about the personality correlates of parental burnout and the role of personality at different stages of parenthood.

## Introduction

Research demonstrates that long-term stress associated with the parental role can lead to parental burnout (Mikolajczak et al., 2019, 2021). This syndrome involves overwhelming exhaustion related to one’s parental role, emotional distancing from children, loss of parental fulfillment, and contrast between the previous and current parental self (Roskam et al., 2018). While previous studies have found that a parent’s psychological characteristics, such as personality traits, play a key role in the occurrence of parental burnout (Le Vigouroux et al., 2017; Vigouroux and Scola, 2018; Mikolajczak et al., 2018b), many issues need further exploration.

First, temperamental traits, which are the basic biological basis for individual differences (Rothbart, 2007), have not yet been studied in the context of parental burnout. Since temperamental traits affect the ability to process stimulation and cope with stress, it seems likely that they also have an impact on the risk of parental burnout. Second, the sense of parental identity (Fadjukoff et al., 2016), which helps cope with parental stress (Piotrowski, 2022a) and provides a buffer to protect the parent from the effects of difficult experiences (Schrooyen et al., 2021; Piotrowski, 2022b), has received little attention in previous studies. A thorough understanding of the parental identity–parental burnout link can be of great scientific and practical importance, as parental identity develops from adolescence, even before an individual becomes a parent (Gyberg and Frisén, 2017), potentially making this characteristic one of the early predictors of parental burnout in the future. Third, recent studies on parental stress suggest that personality traits are more strongly associated with the level of parental stress in the first years of parenthood than when an individual has been in the parenting role for many years (Rantanen et al., 2015). In the present study, we sought to investigate whether this observation might also apply to parental burnout.

For this purpose, we assessed the association of parental burnout with temperament, parental identity, and Big Five traits among people at different stages of parenthood (i.e., a specific period in a parent’s life when they face distinctive challenges and tasks; Rodgers and White, 1993). For the purposes of the study, we distinguished the stages of parenthood based on a criterion often used by family researchers, namely the age of the children in the family (Hill, 1986; Buchanan et al., 2016). Although the age of children is not a strong correlate of parental burnout, researchers systematically report that caring for children in the early childhood or preschool periods is the most demanding (Nelson et al., 2014) and carries a higher risk of burnout (Mikolajczak et al., 2018b). For this reason, it is in the early stages of parenthood that having personality traits that can protect against burnout can be particularly important (see Rantanen et al., 2015). Verification of this hypothesis was one of the objectives of the study.

### Personality determinants of parental burnout

According to McAdams and Pals (2006), personality should be considered as a complex system consisting of characteristics that form successive levels. At the basic level, personality is formed by evolutionarily determined characteristics typical for our species (e.g., categorizing people into “in-group” and “out-group”). At a second level, there are relatively stable dispositional traits responsible for behavior in a wide range of situations, resulting from the interaction of genetic factors and an individual’s experiences. This level of analysis includes temperament traits (genetically determined predispositions upon which personality further develops; Kandler et al., 2012), and the Big Five personality traits (Costa and McCrae, 1992). Finally, at an even higher level, there are characteristic adaptations, i.e., specific patterns of behavior, motivation, and styles of thinking that are the effect of an individual’s experiences in the course of life. At this level, we find such characteristics as a style of coping with stress, emotional intelligence, perfectionism, attachment styles, as well as a sense of identity that was analyzed in the present study (McAdams and Pals, 2006).

To date, research on parental burnout has not examined the role of temperament, which is the primary biological basis of personality (Kandler et al., 2012) associated with the formal (rather than content-related) characteristics of behavior. The study of temperament traits, more so than the study of the Big Five traits, allows for the observation of an organism’s need for stimulation, ability to process strong stimulation, and ability to regulate/supply itself with stimulation at an appropriate level (Rothbart, 2007). A parent’s temperament affects not only the level of experienced stress (Kandler et al., 2012), but also the parenting methods used, and not only among humans, but also among other species (Maestripieri, 1999). Difficulty in processing strong stimulation can lead to frequent feelings of anxiety and restlessness in the parent, leading to a strong focus on the child, overprotection and limiting the child’s exploration (Lee et al., 2015; Bilgiç et al., 2018), which in the long term is also exhausting for the parent him/herself and can lead to burnout (Sorkkila and Aunola, 2020, 2022). Studies in rats, sheep, primates and humans have also shown that the temperamental profile characterized by low emotional resilience, impulsivity and a low response threshold increases the incidence of violence against offspring (Pryce, 1992; Maestripieri, 1999), which we also observe among burned-out parents (Mikolajczak et al., 2018a). For this reason, in the present study, we hypothesized that certain temperamental traits (especially those related to low stimulation processing ability) may increase the risk of parental burnout. The present study is the first in which the temperament–parental burnout link is examined.

In studies of dispositional traits, the Big Five traits have received the most attention to date (Costa and McCrae, 1992). Research has revealed a specific role for two traits: neuroticism, which is positively related to levels of parental burnout, and agreeableness, which is negatively related to burnout (Le Vigouroux et al., 2017; Gérain and Zech, 2018; Roskam et al., 2018; Mikolajczak et al., 2018b; Furutani et al., 2020). Some studies have also found that parental burnout is negatively correlated with extraversion (Szczygieł et al., 2020) and conscientiousness (Vigouroux and Scola, 2018). Overall, these results indicate that traits identified with resilient personality (Caspi et al., 2005), namely low neuroticism and high conscientiousness, agreeableness, and extraversion, are associated with lower parental burnout.

With regard to traits from the level of characteristic adaptations that are more specific and less rooted in biology than temperament or the Big Five traits, there have been a number of studies to date on parental burnout. For example, studies found positive relations between parental burnout and attachment anxiety (Mikolajczak et al., 2018b), prioritizing values related to power and individual achievement (Lin and Szczygieł, 2022), and perfectionistic concerns (Kawamoto et al., 2018), and negative relations with resilience (Sorkkila and Aunola, 2022), trait emotional intelligence (Lin et al., 2022), and prioritizing values related to benevolence (Lin and Szczygieł, 2022). A limitation of most of these studies is that they typically have not controlled for the more basic levels of personality organization with which many characteristic adaptations are correlated. With respect to values, Bojanowska and Piotrowski (2021) observed not only that temperament traits and personal values are correlated, but also that when their joint variance is controlled for, both characteristics are associated with different aspects of quality of life. In the context of burnout research, Mikolajczak et al. (2018b) found that the positive correlation between parental burnout and attachment anxiety and avoidance (Mikulincer and Shaver, 2019) almost completely disappeared when other traits, including Big Five traits, were controlled for in the regression model. On the other hand, the same study found that emotional intelligence remained a significant predictor of burnout even when controlling for basic personality traits. This suggests that controlling for the common variance of various correlates of parental burnout may allow for better identification of specific risk factors.

The characteristic adaptation we considered in the present study was a sense of parental identity, that is, having a stable commitment to the parenting domain and a well-formed definition of oneself as a parent (Fadjukoff et al., 2016; Gyberg and Frisén, 2017). Piotrowski (2021a, 2022b) observed that parents with a poorly formed sense of parental identity are characterized by higher parental burnout. Unfortunately, none of the studies assessed whether the relationship between sense of parental identity and parental burnout remains significant when controlling for more basic levels of personality. A number of previous studies on identity development have shown that individuals who have greater difficulty coping with identity crisis are those with low stimulation processing ability (an important dimension of temperament, Bojanowska and Zalewska, 2017), low conscientiousness and agreeableness, and high neuroticism (Clancy and Dollinger, 1993; Klimstra et al., 2013), which may explain the observations regarding parental identity. One of the goals of the study was to disentangle these characteristics, namely temperament, Big Five traits, and parental identity, and reveal their role for parental burnout.

### Personality and parental burnout at different stages of parenthood

An issue that has not yet been explored is also the potential interaction between a parent’s personality traits and stage of parenting. The birth of the child in the family and its growth confronts parents with new tasks and challenges and affects the degree of burden on the parenting role (Nelson et al., 2014). From the point of view of the changing demands that parents face, several stages of parenthood can be distinguished, including (see Hill, 1986; Buchanan et al., 2016): a *family with a small child/toddler* (up to the age of three), when parents need to integrate the parental role with other roles they perform (when it is the first child) or reorganize family life when another child is born, while at the same time devoting a great amount of time and energy to caring for a child of this age; *a family with a preschool child*, when parents reorganize family life around the child’s growing autonomy and needs, often having to deal with difficult child behavior (Giesbrecht et al., 2010); a *family with a school-age child*, when the family reorganizes as a result of the child’s greater transition into the outside world and the start of formal education; a *family with an adolescent*, when the main task becomes changing the family relationship toward greater independence; and a *family with an adult child*, when the family relationship must evolve toward the equal status of parent and child.

Although fulfilling the role of a parent is often a source of stress regardless of the stage of parenthood (Berry and Jones, 1995), studies show that the burden of parenthood is greatest during the first stages, when early childhood and preschool children are under care (Nelson et al., 2014; Rantanen et al., 2015). Thus, one might speculate that it is in the early stages of parenting (especially before the child reaches school age; Nelson et al., 2014) that it matters most whether a parent has the resources to avoid burnout. As children increase in age and the demands of parenting decrease, or as competence to cope with the demands of parenting increases with time, the importance of particular personality traits (for example, whether a parent has low or high levels of neuroticism) may decrease. This effect in relation to parental stress was demonstrated in a longitudinal study by Rantanen et al. (2015). These authors found that the positive association of neuroticism and the negative association of extraversion with parental stress were stronger when parents were between 33 and 42 years old (the average age of their children was then about 6 years old) than when they were between 42 and 50 years old (their children were on average about 16 years old). Because parental burnout is a consequence of parental stress (Mikolajczak et al., 2019), we predicted that this effect should be similar.

### Research problem and hypotheses

The present study had two main objectives. The first was to assess associations between traits from different levels of personality organization and parental burnout (McAdams and Pals, 2006). We predicted that at each of the levels analyzed (temperament, basic personality traits, and identity) we would observe characteristics associated with burnout scores. At the temperamental level, parental burnout would be associated with low ability to process stimulation, low arousal threshold, and a tendency to respond strongly to stimulation. We hypothesized that these temperamental traits might increase the risk of burnout because parenting is a social role that carries many burdens and responsibilities and therefore also provides strong stimulation that individuals with certain temperamental traits may not be able to handle.

Since temperamental traits such as low ability to process stimulation and emotional reactivity promote the development of neuroticism (Rothbart, 2007), which is one of the key predictors of parental burnout (Le Vigouroux et al., 2017), we predicted that in addition to temperamental traits, we would also observe a specific positive relationship between neuroticism and parental burnout. With regard to the level of characteristic adaptations, we predicted that, independent of temperament and basic personality traits, difficulty in forming a stable definition of oneself as a parent (parental identity) would also be associated with higher parental burnout (Schrooyen et al., 2021; Piotrowski, 2021a,b).

The second goal of the study was to determine whether the analyzed traits interact with the stage of parenthood at which an individual is. Research on parental stress (Rantanen et al., 2015) and the differential effects of personality traits on behavior at different life stages (Wrzus et al., 2016) support this prediction. Hence, we hypothesized that because taking care of children in the early childhood and preschool period requires adaptation to a complex task and demands a high emotional and time commitment (Rantanen et al., 2015; Mikolajczak et al., 2018b), the ability to process strong and dynamically changing stimulation (temperament), emotional stability (basic personality traits), and a clear self-image as a parent and identification with the parenting role (parental identity) may then be more significant for burnout risk than at later stages of parenting.

## Method

### Procedure and participants

The research was conducted online, using the SurveyMonkey platform. Originally, *N* = 1993 participants opened the survey page and answered at least some of the questionnaire questions, but 522 people withdrew from participation at different stages, usually shortly after the beginning. The results of the remaining 1,471 people who reached the end of the survey were included in the analyses. Since answering all questions was mandatory (it was not possible to skip questions), the final database does not contain missing values. The study was positively evaluated by the Departmental Ethics Committee at SWPS University, Poznań, Poland. University students were involved in data collection and recruited participants using their personal contacts. Because students came from all over the country, the research sample was geographically diverse.

The sample included *N* = 1,471 Polish parents, ranging in age from 19 to 45 years (*M* = 35.30, SD = 5.98). There were 1,199 women (81.5%), 265 men (18%), and 7 individuals who identified their gender as nonbinary or did not want to provide gender information (0.5%). Participation was conditional on having at least one child living in the same household. The age of children ranged from 1 month to 26 years (*M* = 8.22, SD = 6.21), and participants had between one (45.5%) and five (0.3%) children. A question about the presence of chronic illnesses and disabilities in children revealed that 13.1% of parents were raising a child with long-term health problems. Of the participants, 73.6% were married, 18.8% were in an informal relationship, and 7.6% were singles. Most individuals had a college education (71.4%). The sample varied by place of residence, with 29.1% living in cities of 500,000 or more, 47.3% living in smaller cities and towns, and 23.7% living in villages.

### Measures

#### Parental burnout

Participants completed the 23-item Parental Burnout Assessment (Roskam et al., 2018; Polish version by Szczygieł et al., 2020). The scale measures different manifestations of parental burnout (e.g., *I feel completely run down by my role as a parent*) and allows for a global score that was used in the analyses. Participants rated each statement on a seven-point Likert scale, ranging from 0-*never* to 6-*daily*. Cronbach’s alpha in the current study was 0.96.

#### Temperament

Temperament traits were measured using a shortened version of the Formal Characteristics of Behaviour—Temperament Inventory (Strelau et al., 1990). The scale consists of 42 items that form six subscales (7 items in each) and allows for measurement of six different temperamental traits: *Briskness*—tendency to react quickly, keep a high tempo and to easily shift from one behavior to another in response to changes in the surroundings (e.g., *I usually do things quickly*, e.g., *house chores, tidying*); *Perseveration*—tendency to continue and to repeat behavior after the stimulus evoking this behavior had stopped acting (e.g., *Before falling asleep, I go back to conversations I had that day*); *Sensory sensitivity*—ability to react to weak sensory stimuli (e.g., *I can even smell the subtle scent of flowers*); *Emotional reactivity*—tendency to react intensely to emotion-generating stimuli, high emotional sensitivity and low emotional endurance (e.g., *I lose confidence when someone criticizes me*); *Endurance*—ability to react adequately to situations that demand long-lasting or highly stimulating activity and under intense external stimulation (e.g., *I am able to keep working despite being tired*); *Activity*—tendency to undertake highly stimulating behavior (e.g., *I organize my holidays in such a way that I get a lot of new experiences*). Briskness and Perseveration are traits that account for the temporal characteristics of behavior and refer to how quickly a response to a stimulus occurs and how long it persists after the stimulus has ended. Sensory sensitivity, Emotional reactivity and Endurance are energetic dimensions that are responsible for the registering and processing of stimulation, while Activity is responsible for regulating the inflow of stimulation by engaging in various tasks. Cronbach’s alphas for each subscale ranged from 0.65 to 0.76, which is in line with other studies using this scale (De Pascalis et al., 2000; Bojanowska and Piotrowski, 2021).

#### Basic personality traits

The Big Five traits were measured using the International Personality Item Pool-Big Five Markers-20 (IPIP-BFM-20; (Topolewska et al., 2014), which is a short version of IPIP-BFM-50 (Donnellan et al., 2006)). It is a 20-item scale for measuring the Big Five traits with four items for each trait (e.g., extroversion: *Am the life of the party*). Each item is assessed on a five-point Likert scale, ranging from 1—*very inaccurate* to 5—*very accurate*. Cronbach’s alpha values were similar to those of the validation study (Topolewska et al., 2014): extroversion 0.82; agreeableness 0.63; conscientiousness 0.77; emotional stability (the opposite of neuroticism) 0.73; and intellect/openness 0.67.

#### Parental identity

The Utrecht-Management of Identity Commitments Scale (U-MICS; Crocetti et al., 2008) in the version for investigating parental identity (Piotrowski, 2018, 2020) was applied to measure three parental identity processes: *commitment*—indicates whether an individual has made important decisions regarding parenting (5 items, e.g., *Being a parent makes me feel sure of myself*); *in-depth exploration*—is a reflective process of seeking in-depth information about commitments made and supports parental identity maintenance (5 items, e.g., *I try to find out a lot about my child/children*); and *reconsideration of commitment*—process of comparing currently made commitments with alternative paths of identity development; high reconsideration of commitment can point to regrets, not accepting parenthood—dreaming of how good it would have been not to become a parent—and thus changing one’s identity (3 items, e.g., *I often think it would have been better not to have had any children*). Investigated individuals would provide their answers on a five-point Likert scale, ranging from 1—*completely untrue* to 5—*completely true*. High commitment and low reconsideration of commitment are taken as indicators of a well-formed parental identity, while in-depth exploration has a supportive function and supports the formation of commitment. Cronbach’s alphas were 0.89, 0.72, 0.90, respectively.

### Statistical analyses

Data analysis was performed using SPSS 26. Analysis of the distribution of variables indicated that the skewness of all variables analyzed was between −2 and 2 (a range of moderate deviation from normality), while kurtosis exceeded the value of 2 only in three cases: sensory sensitivity (kurtosis value of 2.98); reconsideration of commitment (kurtosis value of 3.78); and parental burnout (kurtosis value of 2.57). Since parametric tests remain robust for such distributions, especially for large samples (Edgell and Noon, 1984; Rasch and Guiard, 2004; Schmider et al., 2010), it was decided to use them in the data analysis.

As a first step, *r*-Pearson correlation analysis was conducted to assess the relationship of parental burnout, temperament, Big Five traits, and parental identity. Multiple regression analysis was then used to assess specific associations between parental burnout and demographic characteristics and traits from different personality levels. Demographic variables (gender and age of parent, average age of children and number of children, presence of chronic illness/disability of child) were entered into the regression model in the first step, the Big Five traits were entered in the second step, and temperament traits were entered in the third step. Temperament traits were entered later to assess whether they could explain additional variance in parental burnout relative to Big Five traits. Since the current study is the first to include temperament, it was considered important to determine whether measuring this construct provides additional information. In a final step, dimensions of parental identity were included in the model to test whether they would be significant predictors of parental burnout when demographic characteristics, temperament and Big Five traits were controlled. Taking into account the correlations between some of the predictors (Supplementary Table), we also checked the risk of multicollinearity, which could negatively affect the results of regression analysis. However, it turned out that for none of the predictors did the value of the variance inflation factor (VIF) exceed 2.5, and in most cases the VIF was close to 1, showing that multicollinearity did not affect the results.

To verify the hypothesis on the interaction of personality traits and parenthood stage, it was necessary to divide the study sample into subgroups of participants at different stages. Under a family development model (Hill, 1986; Davies and Gentile, 2012), the life cycle of a family changes with the duration of parenthood, which is determined by the age of the oldest (or only) child. In this case, a differentiation is made between families with the eldest child in early childhood (0–3 years, when parents are adapting to their new role), then a family with a preschool-aged child (in Poland this is age 4–6), with a school-aged child (7–12 years), a family with an adolescent (13–20 years), and finally a family with an adult child (over 20 years). At each stage, parents face different challenges (Hill, 1986), and their personality traits may have different implications for the level of parenting stress experienced (Rantanen et al., 2015). This method of distinguishing parenthood stages was applied in the analysis of the results. The study sample was divided by the age of the oldest/only child into subgroups of 0–3 years (*n* = 417, 28.3%), 4–6 years (*n* = 274, 18.6%), 7–12 years (*n* = 442, 30%) and 13 or more years (*n* = 338, 23%; since the group of parents with an adult child, over 20 years old, was small, *n* = 81, they were pooled with the group of parents of adolescents).

Since focusing on the age of the eldest child does not take into account the changes that the appearance of subsequent children brings to the family (Hill, 1986), the analysis of the results also uses a different approach to distinguish the stages of parenthood. The literature on parental burnout emphasizes that regardless of how long a person has been in the role of a parent, the risk of burnout also increases (although not very strongly) when they have at least one child under the age of five in their care (Mikolajczak et al., 2018b; Furutani et al., 2020). Caring for a small child, also when it is a subsequent child in the family, can thus be considered a burnout-relevant stage of the family life cycle, when having personality resources to counteract burnout may be more important than at a later time. To verify this suggestion, in addition to the division into four subgroups described above, the study sample was also divided into two subgroups, based on the presence of at least one child in the family aged 5 years or less (*n*_yes_ = 886, 60.2%, *n*_no_ = 585, 39.8%). Analysis of variance was then used to test whether parenting stage (distinguished in two ways) and temperament and personality traits interact in relation to scores of parental burnout. For this purpose, all temperamental and personality variables were divided into three categories (low, medium, high level) so that approximately 33% of the results were in each category. The only exception was Reconsideration of commitment, which had small variation and was divided into two categories (low-high score).

## Results

Table 1 presents descriptive statistics and correlations between the study variables and parental burnout. Correlations between all study variables are shown in Supplementary Table.

**Table 1:** Descriptive statistics and correlations between analyzed variables.

	Min	Max	*M*	SD	Parental burnout
Parental burnout	0	131	25.68	24.45	–
*Temperament*					
Briskness	0	1.00	0.74	0.24	−0.32***
Perseveration	0	1.00	0.60	0.25	0.22***
Sensitivity	0	1.00	0.85	0.22	−0.16***
Reactivity	0	1.00	0.49	0.30	0.32***
Endurance	0	1.00	0.53	0.27	−0.36***
Activity	0	1.00	0.39	0.29	−0.19***
*Big Five*					
Extraversion	1.00	5.00	3.22	0.91	−0.20***
Agreeableness	1.00	5.00	3.90	0.67	−0.13***
Conscientiousness	1.00	5.00	3.55	0.89	−0.21***
Emotional Stability	1.00	5.00	2.89	0.81	−0.38***
Intellect/Openness	1.00	5.00	3.75	0.71	−0.16***
*Parental identity*					
Commitment	1.00	5.00	3.52	0.92	−0.43***
In-depth exploration	1.00	5.00	3.96	0.59	−0.11***
Reconsideration	1.00	5.00	1.47	0.78	0.58***

*p < 0.05; **p < 0.01; ***p < 0.001.

To control for the common variance of the study variables, multiple regression analysis was conducted (Table 2). Results revealed that despite many significant bivariate correlations, parental burnout was in specific relationships with only a few of the variables analyzed. Parental burnout was not significantly related to the gender or age of the parent, or the number of children in the family, but was negatively related to the average age of the children, and positively related to the child’s chronic illness/disability. The sociodemographic variables included explained a total of 5% of the variance in parental burnout.

**Table 2:** Results of regression analysis with parental burnout as a dependent variable.

	*β*	*F*	*R* ^2^	Δ*R*^2^
*Step 1*		16.02***	0.05	
Gender (1-female, 2-male)	−0.03			
Age of the parent	−0.01			
Average age of children	−0.20***			
Child’s disability (0-no, 1-yes)	0.13***			
Number of children	0.08			
*Step 2*		42.23***	0.22	0.17***
Extraversion	−0.06			
Agreeableness	−0.07			
Conscientiousness	−0.15***			
Emotional Stability	−0.32***			
Intellect/Openness	−0.06			
*Step 3*		35.67***	0.28	0.06***
Briskness	−0.13***			
Perseveration	−0.01			
Sensitivity	−0.08			
Reactivity	0.02			
Endurance	−0.17***			
Activity	−0.03			
*Step 4*		76.18***	0.49	0.21***
Commitment	−0.16***			
In-depth Exploration	−0.03			
Reconsideration	0.41***			

*p < 0.05; **p < 0.01; ***p < 0.001.

Due to large sample size, significant results were considered to be those whose significance level exceeded *p* < 0.001. Beta values are given for each step of the regression analysis when controlling for previously entered variables.

With respect to the Big Five traits, parental burnout was negatively associated only with Conscientiousness and Emotional Stability, and a total of five traits explained an additional 17% of the variance. As predicted, the inclusion of temperamental traits in the model helped explain an additional variance (6%) in parental burnout which shows that a parent’s temperament explains at least part of the variance in parental burnout that is not explained by the Big Five traits. Burnout severity was found to be negatively related to levels of Briskness and Endurance. Finally, adding the dimensions of parental identity to the model helped explain another 21% of the variance. Burnout was negatively related to Commitment and positively related to Reconsideration of commitment. Level of In-depth exploration was not associated with level of burnout. All variables included in the model explained 49% of the variance in parental burnout.

In the next step, parents at different stages of parenthood were compared in terms of parental burnout. When the level of burnout was compared between groups separated by the age of the oldest child, the differences were found to be statistically significant, *F* (3, 1467) = 16.25, *p* < 0.001, *η*^2^ = 0.03. A *post hoc* test (Tukey HSD) revealed that parental burnout was lower in the 13+ group (*M* = 19.02, SD = 21.04) than in the other groups. Significantly higher burnout was observed in the 0–3 (*M* = 27.33, SD = 25.30) and 7–12 (*M* = 25.09, SD = 22.92) groups, and the highest score occurred in the group with a preschool-aged child, 4–6 (*M* = 32.31, SD = 27.31). There was also a significant difference between parents raising at least one child under the age of five and those who had only older children, *F* (3, 1469) = 33.58, *p* < 0.001, *η*^2^ = 0.02. The former group revealed higher levels of burnout (*M* = 28.65, SD = 25.31) than the latter (*M* = 21.18, SD = 22.38).

Next, several two-factor analyses of variance were conducted, with the parenthood stage and different temperament/personality traits, to examine whether trait level was differentially related to burnout level in each subgroup. Each trait was separately included in the analysis to test its interaction with parenthood stage. For most traits, no significant interaction was observed, which suggests that the associations of the studied traits with parental burnout tended to be similar across different subgroups. In several cases, however, a significant interaction of factors was observed.

**Figure 1:**
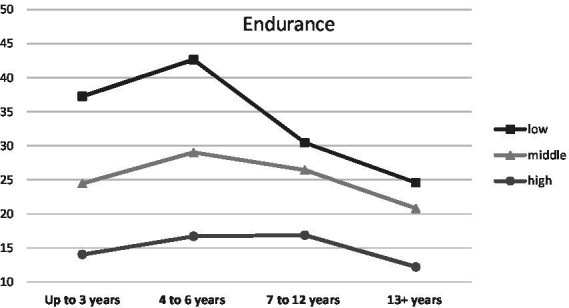
Endurance and parental burnout at different stages of parenting.

**Figure 2:**
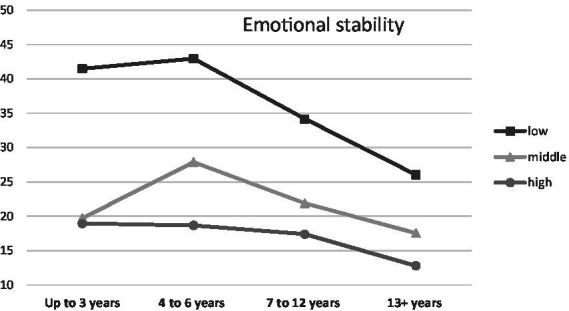
Emotional Stability and parental burnout at different stages of parenting.

**Figure 3:**
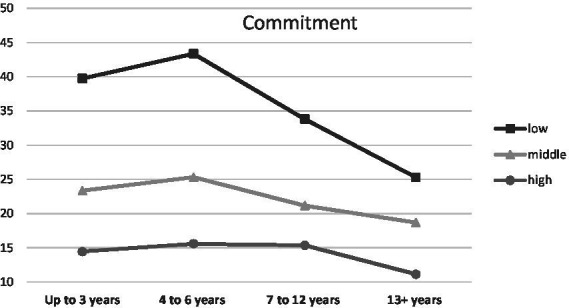
Commitment and parental burnout at different stages of parenting.

**Figure 4:**
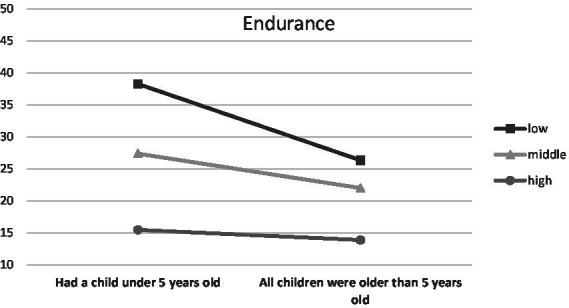
Endurance and parental burnout at different stages of parenting.

**Figure 5:**
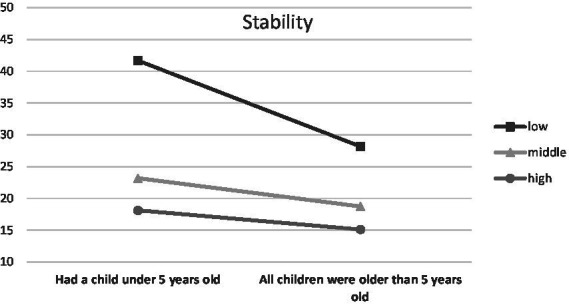
Emotional Stability and parental burnout at different stages of parenting.

**Figure 6:**
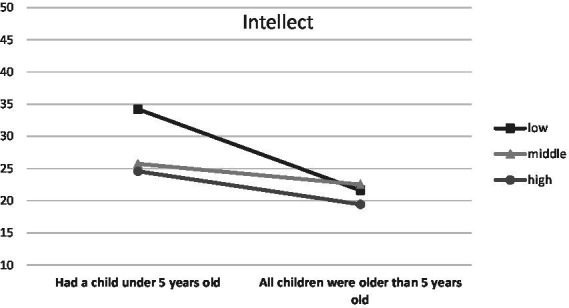
Intellect/Openness and parental burnout at different stages of parenting.

When parenthood stages were separated by the age of the oldest/only child, an interaction was observed with Endurance (temperamental trait; low scores between 0 and 0.43, middle scores between 0.44 and 0.71, high scores between 0.72 and 1.00), *F* (6, 1459) = 3.48, *p* < 0.01, *η*^2^ = 0.01 (Figure 1), Emotional Stability (trait of the Big Five; low scores between 1 and 2.50, middle scores between 2.51 and 3.25, high scores between 3.26 and 5.00), *F* (6, 1459) = 2.66, *p* < 0.05, *η*^2^ = 0.01 (Figure 2) and Commitment (parental identity dimension; low scores between 1 and 3.20, middle scores between 3.21 and 4.00, high scores between 4.01 and 5.00), *F* (6, 1459) = 2.67, *p* < 0.05, *η*^2^ = 0.01 (Figure 3). In contrast, for parents with or without children under the age of five, significant interactions were observed for Endurance, *F* (2, 1465) = 7.65, *p* < 0.01, *η*^2^ = 0.01 (Figure 4), Emotional Stability, *F* (2, 1465) = 7.72, *p* < 0.01, *η*^2^ = 0.01 (Figure 5), and Intellect/Openness (low scores between 1 and 3.50, middle scores between 3.51 and 4.00, high scores between 4.01 and 5.00), *F* (2, 1465) = 5.33, *p* < 0.01, *η*^2^ = 0.01 (Figure 6).

All obtained interactions revealed a consistent picture (Figures 1–6). The interactions of the studied factors consisted of two main effects. First, differences in parental burnout between parents at different stages of parenting were significant almost exclusively among those with low Endurance, low Emotional Stability, low Commitment, and low Intellect/Openness. Results revealed that during the 0–3 and 4–7 years after the birth of the first/only child, low Endurance, low Emotional Stability, low Commitment were associated with higher burnout than among parents at later stages of parenting. Second, differences between parents with different levels of Endurance, Emotional Stability, and Commitment were higher at 0–3 and 4–7 than at 8–12 and over 13+ years. Similar relationships occurred for those caring for a child under the age of five.

## Discussion

We examined the relationship between parental burnout and characteristics from different levels of personality organization (McAdams and Pals, 2006), including for the first time the role of temperament traits in predicting parental burnout. We also examined the hypothesis that the relationship between personality traits and parental burnout will be different depending on the stage of parenthood. Specifically, we predicted that differences between parents with different levels of a given trait will be greater when they are in the early stages of parenthood than when they have been parents longer or their children are older.

Overall, the results obtained support the hypotheses and were in line with predictions, and the findings of other researchers (e.g., Mikolajczak et al., 2018b; Furutani et al., 2020). Parental burnout has indeed been found to be related to traits from all levels studied, including the temperament of the parent. The results also suggest that having personality traits that reduce the risk of burnout may be particularly important during the first six years of parenthood (before the first child reaches school age).

Taken together, the results suggest that such parental traits as low ability to perform under strong stimulation (Endurance), emotional lability and a tendency to react with negative emotions (Emotional Stability/Neuroticism), closeness to new ways of behaving and ideas (Openness), and a poorly formed definition of oneself as a parent (Commitment) are particularly strong risk factors in the period before the child reaches school age. At subsequent stages of parenthood, the importance of these traits may be somewhat lower, although it is worth noting that their effect has not disappeared for parents of teenagers and even adult children. In sum, our observations are consistent with the results of the study by Rantanen et al. (2015) and confirm that the effect observed earlier for parental stress is also present for parental burnout. Our study also shows that this does not only apply to the Big Five traits, but also to other personality characteristics associated with parental burnout.

### The role of temperament in parental burnout

In terms of the effects of temperament, the data showed that higher Briskness and Endurance were associated with lower burnout. Higher Briskness allows people to shift easily between tasks—disengage from one task and switch onto another one (Strelau et al., 1990). This ability promotes better coping with versatile tasks that are typical of a parenting situation. People with higher Briskness generally have higher indices of well-being (Bojanowska and Zalewska, 2017), they probably cope with challenges better and our data show that this extends also (inversely) to parental burnout. The observed effect of Endurance is consistent with its function described in the Regulative Theory of Temperament (Strelau et al., 1990). This trait is responsible for coping with high stimulation and the ability to keep engaged in tasks despite strong stimulation. More enduring parents can probably cope better with issues such as lack of sleep, a multitude of different tasks and the fact that there is sometimes little option to disengage and recover. All this translates into lower burnout. Additionally, high Endurance seemed to shield from burnout especially at the earlier stages of parenting (when the child is in the early childhood or preschool period), when fulfilling the role of a parent is still very demanding in terms of time and emotion (Nelson et al., 2014). Our study is the first in which the important role of temperament was revealed in the context of parental burnout. The results indicate that research on personality risk factors should be shifted to a lower level, related to the nervous system arousability and ability to process stimulation. The capabilities of most personality models, even as universal as the Big Five, are limited in this context, warranting further study of the importance of temperament for parental burnout.

### The role of the Big Five traits in parental burnout

Results for the Big Five traits were consistent with expectations and previous research (Le Vigouroux et al., 2017). The most important of the basic personality traits was found to be Emotional Stability (the inverse of Neuroticism), associated with a tendency to respond with positive emotions to events that occur and a lack of frequent mood changes. This is a trait that has a strong influence on parent adjustment (Roskam et al., 2018). Our study also showed for the first time that the importance of Emotional Stability for parental burnout may be higher when a parent cares for a child up to the age of six, during early childhood or the preschool period. When a child enters school age (around seven years old) and then adolescence (around 12/13 years old), differences between parents with low and high Emotional Stability were no longer so pronounced. Similar results were found for Intellect/Openness, the high intensity of which seems to be more important during the first years of parenthood. This may occur because the longer an individual fulfills the role of a parent, the more factors may determine the degree of burnout (e.g., changes in the relationship with the partner), and the parent’s competence may be higher, so the importance of personality traits may diminish. In addition, as children grow older, the intensity of parenthood, including the amount of time spent, decreases, which may make the risk of burnout somewhat lower even in the absence of personality resources. Nevertheless, although diminished, the impact of Emotional Stability on parental burnout does not disappear even after many years of fulfilling this role, remaining a key predictor of difficulties experienced by parents. In addition to Emotional Stability, Conscientiousness was also significantly associated with burnout, which has also been observed previously (Le Vigouroux et al., 2017). More conscientious parents are better organized, plan ahead, are more disciplined and have better time management. These characteristics make it easier for them to fulfill this role and balance it with other responsibilities, contributing to lower stress (Oliver et al., 2009; Prinzie et al., 2009). The lack of interaction of conscientiousness with parenting stage suggests that it is a characteristic that appears protective throughout the parental life.

### The role of parental identity in parental burnout

Although temperament and basic personality traits have been shown to be significantly related to parental burnout, our study shows that it is also important to take into account more specific personality characteristics. Among these, Piotrowski (2022a,b) and Schrooyen et al. (2021) highlighted a sense of parental identity as a factor that protects the parent from negative emotions and stress. Indeed, we found that parental identity dimensions accounted for a similar proportion of the explained variance in parental burnout as temperament and the Big Five traits combined. As with Endurance, Emotional Stability and Openness, we observed that the greatest importance of parental identity may take place before a child reaches school age. However, regardless of the duration of parenting and the age of the children, parents with a well-formed identity were less likely to report parental burnout symptoms. Research on the role of parental identity in parental burnout is important because unlike temperament and basic personality traits, which change slowly, a sense of identity is more dynamic and susceptible to external influences (Berman et al., 2008). However, further studies are needed to test the direction of this relationship, as it can be predicted that parental identity may protect against burnout (Schrooyen et al., 2021), but also that parental burnout may alter the sense of identity (Roskam et al., 2022).

### Practical implications

In addition to encouraging further scientific research on the relationship between personality and parental burnout at different stages of parenthood, the results of our study may also be useful in planning support for parents. Since parenthood stress and parental burnout are difficulties faced by parents all over the world (Roskam et al., 2021), specialists in different fields, physicians, psychologists, social workers, teachers, may encounter them relatively often. Our study shows that those parents who have difficulty functioning in a stimulating environment and tend to experience negative emotions require particular support. Since these are characteristics that are difficult to modify, it may be good practice to support the development of protective factors that can attenuate the negative impact of risk factors. Among such characteristics, for example, is emotional intelligence, which can be developed through intervention programs (Platsidou and Tsirogiannidou, 2016). As a recent study by Lin et al. (2022) showed, high emotional intelligence may protect perfectionistic parents from burnout. One might assume that the same would be true for risk factors such as those revealed in our study, such as high neuroticism. Also, the parental identity we studied, which is an important resource for protecting parents from stress and burnout (Schrooyen et al., 2021; Piotrowski, 2022b) can probably be developed through psychological interventions (Berman et al., 2008; Meca et al., 2014). A study by Brianda et al. (2020) confirmed the high effectiveness of group interventions for parental burnout, showing that although parental burnout is most strongly associated with relatively stable personality traits (Vigouroux and Scola, 2018), well-planned psychological interventions provide important support for parents and reduce parental stress. As our study shows, specialized support may be needed primarily by parents raising young children who have only recently started the parenthood role. In the case of such individuals, we may have to deal with an overlap of various burdens, which requires specialists to take action focused on many areas. Young parents not only have to cope with raising young children, but at the same time complete a number of other important developmental tasks, such as developing a career, building an intimate relationship, and gaining emotional and financial autonomy from the family of origin (Arnett et al., 2014; Mehta and Arnett, 2023). Each of these tasks separately represents a significant source of stress, but for young adults these burdens overlap. Given this situation, it is not surprising that adolescents and young adults in the contemporary world are particularly vulnerable to mental health problems (Xiong et al., 2020). Young parents are no exception (Plass-Christl et al., 2017) and it is they who may be particularly vulnerable to parental burnout and should become the focus of special attention from specialists in different fields.

### Limitations

Despite providing new knowledge about parental burnout risk/protective factors, the present study has several limitations. First, the study sample consisted primarily of women, and although gender was controlled for in part of the analyses, further research with a more balanced sample is needed. Second, the results are from a cross-sectional study and need to be verified in a longitudinal study. Third, all data are from self-report and confirmation by studies using other indicators is desirable. Fourth, the reliability of some of the subscales used was quite low, between 0.60 and 0.70. In future studies, it would be worthwhile to use questionnaires with greater internal consistency. Fifth, in the current study, certain topics were addressed for the first time (e.g., the role of temperament traits, the interaction of personality and parenthood stage), which requires their confirmation in subsequent studies, especially those conducted in countries other than Poland.

## Conclusion and directions for further research

Despite its limitations, our study contributes to research on the personality correlates of parental burnout. First, the results suggest that parental burnout is related to personality characteristics from different levels of personality organization (McAdams and Pals, 2006), beginning with the temperament traits, which affect the ability to act effectively in a stimulating environment, through such basic personality traits as conscientiousness and emotional stability responsible for coping with daily tasks and experienced emotions, to such specific traits as the clarity of one’s definition of oneself as a parent. It is plausible that in different individuals, different personality levels may be the dominant force in the development of parental burnout. Future studies should verify this hypothesis.

Second, the results of the present study suggest that although a parent’s personality is a key correlate of burnout at any stage of parenthood, its impact as a protective/risk factor may be most pronounced in the early stages. Since raising a young child, in early childhood or the preschool period, is the most stressful and burdening (Berry and Jones, 1995; Nelson et al., 2014; Rantanen et al., 2015), this is when parents with low personality resources are most likely to experience severe burnout. In turn, when the child is older and more independent (starting from the school age; Nelson et al., 2014), the importance of personality traits may be somewhat lower, probably because parenthood becomes less intense and burdening, thus requiring less buffering (moderating) mechanisms. Future studies should verify this hypothesis and explore stage-specific protective and risk factors related to parental burnout. Additionally, a promising direction for future longitudinal studies on parental burnout is to analyze this phenomenon from a life course perspective and better understand the dynamics of burnout over the long term.

## Data availability statement

The raw data supporting the conclusions of this article will be made available by the authors, without undue reservation.

## Ethics statement

The studies involving human participants were reviewed and approved by Departmental Ethics Committee at SWPS University, Poznań, Poland. The patients/participants provided their written informed consent to participate in this study.

## Author contributions

KP: conceptualization, methodology, data collection, formal analysis, and writing manuscript. AB: conceptualization and writing manuscript. DS, MM, and IR provided critical revision on the first draft of the manuscript. All authors contributed to the article and approved the submitted version.

## Funding

The work of KP was funded by the National Science Centre in Poland, grant no. 2021/42/E/HS6/00120.

## Conflict of interest

The authors declare that the research was conducted in the absence of any commercial or financial relationships that could be construed as a potential conflict of interest.

## Publisher’s note

All claims expressed in this article are solely those of the authors and do not necessarily represent those of their affiliated organizations, or those of the publisher, the editors and the reviewers. Any product that may be evaluated in this article, or claim that may be made by its manufacturer, is not guaranteed or endorsed by the publisher.
