# Supplementary material for: Parental burnout at different stages of parenthood: Links with temperament, Big Five traits, and parental identity
Source: Front Psychol. 2023 Mar 31;14:1087977. doi: 10.3389/fpsyg.2023.1087977 (PMC10102599; doi:10.3389/fpsyg.2023.1087977)
Supplement: Supplementary file 1 [file Table_1.DOCX]

Supplementary Table

*Correlations between study variables*

|  |  | 1 | 2 | 3 | 4 | 5 | 6 | 7 | 8 | 9 | 10 | 11 | 12 | 13 | 14 | 15 |
| --- | --- | --- | --- | --- | --- | --- | --- | --- | --- | --- | --- | --- | --- | --- | --- | --- |
| 1. | Parental burnout | - | -.32*** | .22*** | -.16*** | .32*** | -.36*** | -.19*** | -.20*** | -.13*** | -.21*** | -.38*** | -.16*** | -.43*** | -.11*** | .58*** |
| 2. | Briskness |  | - | -.28*** | .13*** | -.42*** | .41*** | .18*** | .26*** | .11*** | .24*** | .26*** | .24*** | .17*** | .03 | -.18*** |
| 3. | Perseveration |  |  | - | .04 | .57*** | -.33*** | -.12*** | -.19*** | .07** | -.08** | -.49*** | -.14*** | -.06* | .20*** | .12*** |
| 4. | Sensitivity |  |  |  | - | -.06* | .09*** | .06* | .08** | .18*** | .12*** | .06* | .18*** | .07** | .13*** | -.12*** |
| 5. | Reactivity |  |  |  |  | - | -.44*** | -.21*** | -.35*** | -.05 | -.15*** | -.54*** | -.36*** | -.11*** | .14*** | .17*** |
| 6. | Endurance |  |  |  |  |  | - | .16*** | .16*** | .05 | .19*** | .36*** | .19*** | .21*** | -.02 | -.19*** |
| 7. | Activity |  |  |  |  |  |  | - | .55*** | .26*** | -.01 | .21*** | .20*** | .21*** | .10*** | -.07** |
| 8. | Extraversion |  |  |  |  |  |  |  | - | .29*** | .05 | .25*** | .30*** | .19*** | .06* | -.12*** |
| 9. | Agreeableness |  |  |  |  |  |  |  |  | - | .09*** | .03 | .16*** | .10*** | .17*** | -.18*** |
| 10. | Conscientiousness |  |  |  |  |  |  |  |  |  | - | .12*** | -.03 | .17*** | .09*** | -.16*** |
| 11. | Emotional Stability |  |  |  |  |  |  |  |  |  |  | - | .23*** | .19*** | -.16*** | -.21*** |
| 12. | Intellect/Openness |  |  |  |  |  |  |  |  |  |  |  | - | .02 | .01 | -.06* |
| 13. | Commitment |  |  |  |  |  |  |  |  |  |  |  |  | - | .29*** | -.42*** |
| 14. | In-depth exploration |  |  |  |  |  |  |  |  |  |  |  |  |  | - | -.17*** |
| 15. | Reconsideration |  |  |  |  |  |  |  |  |  |  |  |  |  |  | - |

* *p* < .05, ** *p* < .01, *** *p* < .001
